# Use of methotrexate and risk of skin cancer: a nationwide case–control study

**DOI:** 10.1038/s41416-023-02172-7

**Published:** 2023-02-04

**Authors:** Sam Polesie, Martin Gillstedt, Sigrún Alba Jóhannesdóttir Schmidt, Alexander Egeberg, Anton Pottegård, Kasper Kristensen

**Affiliations:** 1 Department of Dermatology and Venereology, Institute of Clinical Sciences, Sahlgrenska Academy, University of Gothenburg, Gothenburg, Sweden; 2 Region Västra Götaland, Sahlgrenska University Hospital, Department of Dermatology and Venereology, Gothenburg, Sweden; 3 Department of Clinical Epidemiology, Aarhus University Hospital, Aarhus, Denmark; 4 Department of Dermatology, Aarhus University Hospital, Aarhus, Denmark; 5 Department of Dermatology, Bispebjerg Hospital, University of Copenhagen, Copenhagen, Denmark; 6 Clinical Pharmacology, Pharmacy and Environmental Medicine, Department of Public Health, University of Southern Denmark, Odense, Denmark

**Keywords:** Melanoma, Basal cell carcinoma, Squamous cell carcinoma, Epidemiology

## Abstract

**Background:**

Methotrexate (MTX) use has been suspected of increasing the risk of skin cancer. The aim of this investigation was to examine the association between the use of MTX and the risk of basal cell carcinoma (BCC), cutaneous squamous cell carcinoma (cSCC) and cutaneous malignant melanoma (CMM).

**Methods:**

In a nationwide Danish case–control study, we identified incident, histologically verified cases of BCC (*n* = 131,447), cSCC (*n* = 18,661) or CMM (26,068) from 2004 to 2018. We matched 10 controls to each case on sex and birth year using risk-set sampling and computed crude and adjusted odds ratios (ORs) using conditional logistic regression for the use of MTX (≥2.5 g) compared with never-use.

**Results:**

Use of MTX was associated with increased risk of BCC, cSCC and CMM with adjusted ORs of (95% confidence interval) 1.29 (1.20–1.38), 1.61 (1.37–1.89) and 1.35 (1.13–1.61), respectively. For BCC and cSCC, ORs increased with higher cumulative doses. When restricting the study population to patients with psoriasis, the ORs were 1.43 (1.23–1.67), 1.18 (0.80–1.74) and 1.15 (0.77–1.72), respectively.

**Conclusions:**

We observed an increased risk of BCC and cSCC associated with the use of MTX with evidence of a dose–response pattern; however, the association was not consistent when restricting the study population to patients with psoriasis.

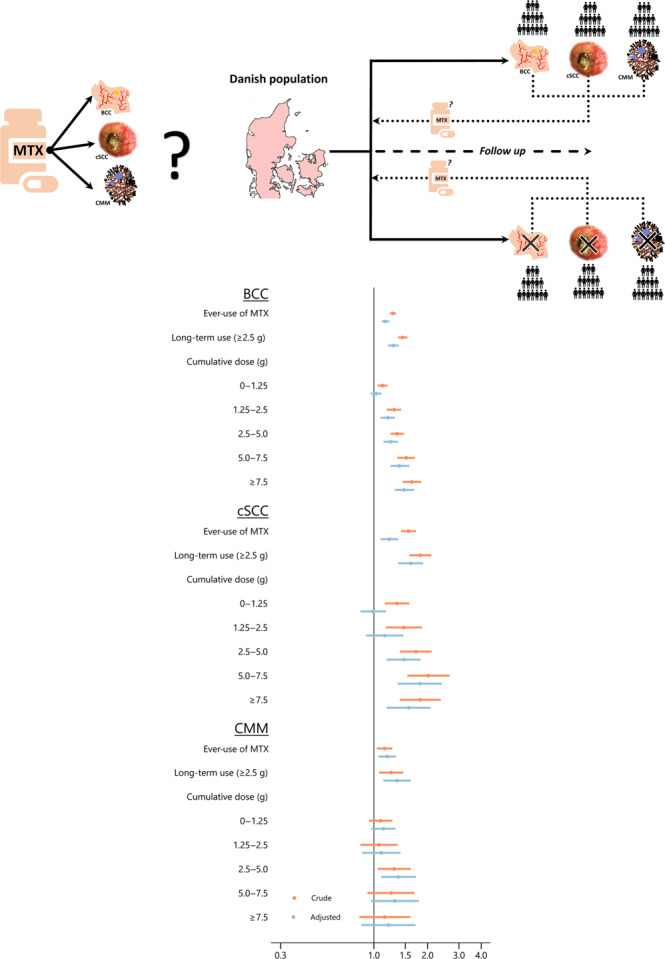

## Introduction

Introduction of biologics has changed the treatment landscape for autoimmune and inflammatory diseases during the past two decades. However, methotrexate (MTX) remains an anchor drug in the dermatological and rheumatological treatment armamentarium. Due to its early introduction in the 1950s, MTX has to a large extent escaped the thorough clinical trials required for contemporary drug approvals [1]. The Cardiovascular Inflammation Reduction Trial (CIRT), a double-blinded prospective clinical trial, investigated if MTX protected against recurrent cardiovascular disease [2]. While no difference in risk of a subsequent cardiovascular event was reported, an increased risk of skin cancer was observed in the MTX-treated group (2.2%) compared with placebo (1.1%) with a hazard ratio (HR) of 2.1 (95% confidence interval [CI] 1.3–3.3) [3, 4].

MTX has immunosuppressive effects and has been linked with photosensitising properties [5, 6], of which both are associated with skin cancer. MTX is listed in the WHO essential list of medicines [7], and considering the relatively high prevalence of MTX users worldwide, an increased risk of skin cancer could have important public health implications. Notably, CIRT was not powered for detecting a difference in skin cancers, had short follow-up (median 2.3 years), and did not reflect clinical prescribing practice for MTX as patients with chronic inflammatory diseases were not eligible. To address these limitations, we carried out a nationwide case–control study on all basal cell carcinoma (BCC), cutaneous squamous cell skin cancer (cSCC), and cutaneous malignant melanoma (CMM) cases in Denmark from 2004 to 2018.

## Methods

We used Danish health and demographic registries to identify all cases of BCC, cSCC and CMM and compared their MTX use with that of matched population controls to obtain odds ratios (ORs) for MTX associated with BCC, cSCC and CMM.

### Data sources

The Danish registries and databases used for this study have been comprehensively described in previous investigations [8–11], and have briefly been described in the supporting material (Appendix S1).

### Study population

We identified cases as patients with a histologically verified first-time diagnosis of BCC, cSCC (excluding squamous cell carcinoma in situ) or CMM (excluding in situ melanoma) from 2004 through 2018. We identified cases from the Danish Cancer Registry [12], using codes from the International Classification of Diseases, Tenth Revision (ICD-10) and the International Classification of Diseases for Oncology, third revision (ICD-O-3) (Supplementary Table 1). We required that on the diagnosis (index) date, participants were ≥18 years of age, had no history of previous cancer (except non-melanoma skin cancer [NMSC] for CMM cases), organ transplantation or HIV-infection, and had been Danish residents for ten years. We matched up to ten Danish residents as controls to each case on birth year and sex using risk-set sampling. Controls were assigned an index date corresponding to the cancer diagnosis date of their case and the same inclusion and exclusion criteria were applied to controls. With this sampling scheme, the ORs are direct estimates of incidence rate ratios (IRR) from a cohort study of the entire Danish population [13]. Since both skin cancer and certain indications for MTX (especially psoriasis and psoriatic arthritis) are more frequent among persons of Nordic origin (typically of fair skin types ranging from I to III), we included only participants originating from any of the Nordic countries (i.e., Denmark, Norway, Iceland, Sweden and Finland).

### Exposure

We collected data on MTX exposure from the Danish National Prescription Registry and the Danish National Patient Registry [14, 15]. The Danish National Prescription Registry contains data on all filled prescriptions at community pharmacies from 1995 and onwards (including dose, volume, and mode of administration), and covered 75–99% of the total sales of MTX in Denmark each year from 1995 to 2018 [16]. The remaining MTX sales stemmed from administrations in the hospital setting, which are not captured in the Prescription Registry. Hospital MTX dispensing were, at least partly, recorded in the Danish National Patient Registry from 1999 and was identified using procedure codes (Supplementary Table 1). We assumed that each hospital dispensing of MTX represented a cumulative dose of 225 mg corresponding to ~3 months of treatment.

Our main exposure was pragmatically defined as a cumulated dose of ≥2.5 grams, which corresponds to 2.7 years of treatment at a maintenance dose of 17.5 mg MTX per week (2.5 mg per day). Further, we examined cumulative dose as an ordinal variable: 0–1.25; 1.25–2.5; 2.5–5; 5–7.5 and ≥7.5 g. Since recent MTX exposure is unlikely to increase cancer risk, we introduced a lag time by disregarding dispensings in the year before the index date.

### Covariates

Potential confounders included (i) age, sex and calendar time (accounted for by study design); (ii) ever-use of drugs with photosensitising properties, including hydrochlorothiazide, oral and topical retinoids, antibiotics (tetracycline, macrolides, fluor- and aminoquinolines), psoralen plus ultraviolet A photochemotherapy (PUVA) [9, 17–19] (iii) exposure to other selective immunosuppressive drugs, including cyclosporine, azathioprine, sulfasalazine, and leflunomide; (iv) exposure to biologic treatment with tumour necrosis factor α inhibitors (TNFis) or interleukin pathway inhibitors (ILis); (v) history of diabetes mellitus, kidney disease, chronic obstructive pulmonary disease, ischaemic heart disease or congestive heart failure, peptic ulcers (including gastric and duodenal ulcers), and alcohol-associated conditions and (vi) highest achieved education. We used ICD-10 discharge diagnoses, procedure codes, and filled prescriptions for drugs commonly used to treat these conditions to define the above covariates (Supplementary Table 1). Educational level was identified from the Danish Education Registries through Statistics Denmark [20]. For all covariates a one-year lag time was applied as for MTX use. To evaluate the importance of each of the potential confounders, we estimated ORs adjusted for each confounder individually, OR for each confounder associated with the outcome, and prevalence of the confounder in MTX exposed and unexposed controls.

### Statistical analyses

We used conditional logistic regression to estimate OR with 95% CIs for each skin cancer associated with the use of MTX in minimally- and fully adjusted analyses. The minimally adjusted (crude) analyses adjusted for age, sex, and calendar time by design, whereas the fully adjusted analyses included all listed covariates above. We evaluated the presence of a dose–response association by including cumulative dose as an ordinal variable in the regression model. In all analyses, we analysed BCC, cSCC and CMM separately and never-use of MTX was the reference group.

To evaluate effect measure modification (heterogeneity) according to specific patient/skin cancer characteristics, we computed fully adjusted ORs in subgroups of sex, age (<65, 65–75, >75 years), and tumour localisation (head and neck, trunk, upper limb, lower limb, other/unspecified) by including an interaction term for the subgroup in the conditional logistic regression model. We estimated *P* values for interaction using a likelihood ratio test of the model without interaction terms nested in the model with interaction terms.

To reduce misclassification due to left censoring (the prescription registry was initiated in 1995), we conducted an analysis restricted to new users by excluding patients that filled any MTX prescription in the time period 1995–1996.

To examine whether the choice of lag time influenced the results, we varied the lag time (i.e., the period before the index date where exposure was disregarded) used to define MTX exposure from 0 to 60 months in 6-month intervals.

To evaluate whether our findings were susceptible to surveillance bias, we repeated the risk-set sampling and analysis restricted to individuals with psoriasis or psoriatic arthritis (defined as a hospital diagnosis of psoriasis, psoriatic arthritis, or a filled prescription of drugs with the ATC code D05AX) before the index date [21].

We performed all analyses using STATA Release 17.0, StataCorp, Texas. The study was approved by the University of Southern Denmark and according to Danish law, ethical approval is not required for registry-based studies.

## Results

After exclusions, we identified 131,447 cases of BCC, 18,661 cases of cSCC, and 26,068 patients with CMM (Supplementary Fig. 1). The median age at diagnosis was 67 years among patients with BCC, 76 years among cSCC patients and 60 years among patients with CMM. Detailed demographic data among cases and controls are presented in Table 1.

**Table 1:** Characteristics of cases and controls.

	BCC		cSCC		CMM	
	Cases	Controls	Cases	Controls	Cases	Controls
	(*n* = 131,447)	(*n* = 1,314,444)	(*n* = 18,661)	(*n* = 186,598)	(*n* = 26,068)	(*n* = 260,680)
Age, years median (IQR)	67 (57–76)	67 (57–76)	76 (68–84)	76 (68–84)	60 (46–71)	60 (46–71)
Male sex	61,466 (46.8%)	614,644 (46.8%)	10,368 (55.6%)	103,674 (55.6%)	11,923 (45.7%)	119,230 (45.7%)
Use of methotrexate						
Ever-use	2816 (2.1%)	22,146 (1.7%)	497 (2.7%)	3224 (1.7%)	451 (1.7%)	3944 (1.5%)
≥2.5 g	1214 (0.9%)	8389 (0.6%)	233 (1.2%)	1296 (0.7%)	183 (0.7%)	1465 (0.6%)
Drug use						
Retinoids	2485 (1.9%)	17,284 (1.3%)	314 (1.7%)	1508 (0.8%)	715 (2.7%)	5970 (2.3%)
Photosensitising antibiotics	77,671 (59.1%)	711,432 (54.1%)	10,886 (58.3%)	98,902 (53.0%)	15,292 (58.7%)	146,462 (56.2%)
Hydrochlorothiazide	19,269 (14.7%)	186,648 (14.2%)	5015 (26.9%)	33,757 (18.1%)	3232 (12.4%)	28,796 (11.0%)
PUVA treatment	173 (0.1%)	1531 (0.1%)	44 (0.2%)	201 (0.1%)	31 (0.1%)	266 (0.1%)
Leflunomide	111 (0.1%)	850 (0.1%)	19 (0.1%)	101 (0.1%)	15 (0.1%)	156 (0.1%)
Azathioprine	1234 (0.9%)	8260 (0.6%)	385 (2.1%)	1101 (0.6%)	178 (0.7%)	1642 (0.6%)
Cyclosporine	194 (0.1%)	1382 (0.1%)	52 (0.3%)	164 (0.1%)	20 (0.1%)	279 (0.1%)
Sulfasalazine	2212 (1.7%)	18,246 (1.4%)	370 (2.0%)	2621 (1.4%)	329 (1.3%)	3288 (1.3%)
TNFis or ILis	519 (0.4%)	3443 (0.3%)	78 (0.4%)	385 (0.2%)	95 (0.4%)	835 (0.3%)
Medical history						
Psoriasis and/or psoriatic arthritis	5062 (3.9%)	42,631 (3.2%)	772 (4.1%)	5985 (3.2%)	831 (3.2%)	8236 (3.2%)
Rheumatoid arthritis	2158 (1.6%)	17,577 (1.3%)	405 (2.2%)	2820 (1.5%)	322 (1.2%)	2889 (1.1%)
Inflammatory bowel disease	1875 (1.4%)	14,837 (1.1%)	290 (1.6%)	2085 (1.1%)	322 (1.2%)	3071 (1.2%)
Atopic dermatitis	230 (0.2%)	2117 (0.2%)	45 (0.2%)	180 (0.1%)	49 (0.2%)	754 (0.3%)
Unspecified dermatitis	520 (0.4%)	5140 (0.4%)	105 (0.6%)	826 (0.4%)	97 (0.4%)	983 (0.4%)
Alcohol-associated conditions	4203 (3.2%)	58,375 (4.4%)	667 (3.6%)	6,819 (3.7%)	791 (3.0%)	12,127 (4.7%)
Diabetes	9083 (6.9%)	111,248 (8.5%)	2290 (12.3%)	20,032 (10.7%)	1695 (6.5%)	18,325 (7.0%)
COPD	6806 (5.2%)	78,533 (6.0%)	1754 (9.4%)	15,152 (8.1%)	863 (3.3%)	11,873 (4.6%)
Kidney disease	1586 (1.2%)	16,356 (1.2%)	588 (3.2%)	3664 (2.0%)	271 (1.0%)	2637 (1.0%)
Peptic ulcer	3043 (2.3%)	34,704 (2.6%)	784 (4.2%)	7089 (3.8%)	440 (1.7%)	5313 (2.0%)
Ischaemic heart disease or congestive heart failure	14,964 (11.4%)	152,798 (11.6%)	3525 (18.9%)	32,704 (17.5%)	2171 (8.3%)	23,250 (8.9%)
Education						
Short	37,048 (28.2%)	462,586 (35.2%)	7131 (38.2%)	74,332 (39.8%)	6195 (23.8%)	78,663 (30.2%)
Medium	58,036 (44.2%)	547,429 (41.6%)	6903 (37.0%)	66,548 (35.7%)	12,261 (47.0%)	116,880 (44.8%)
Long	31,484 (24.0%)	253,243 (19.3%)	2818 (15.1%)	27,473 (14.7%)	6963 (26.7%)	58,108 (22.3%)
Unknown	4879 (3.7%)	51,186 (3.9%)	1809 (9.7%)	18,245 (9.8%)	649 (2.5%)	7029 (2.7%)

*BCC* basal cell carcinoma, *COPD* chronic obstructive pulmonary disease, *ILis* interleukin pathway inhibitors, *IQR* interquartile range, *cSCC* cutaneous squamous cell carcinoma, *CMM* cutaneous malignant melanoma, *OR* odds ratio, *PUVA* psoralen plus ultraviolet A photochemotherapy, *TNFis* tumour necrosis factor α inhibitors.

### BCC

**Fig. 1:**
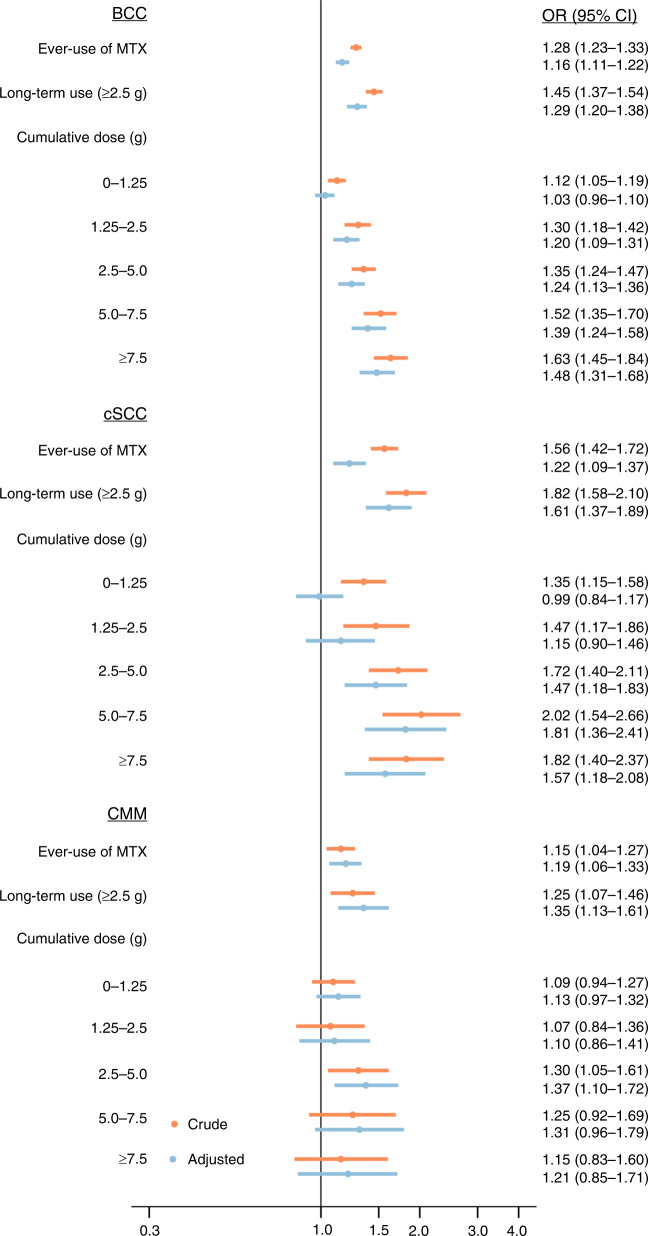
Risk of basal cell carcinoma, cutaneous squamous cell carcinoma, and cutaneous malignant melanoma according to cumulative methotrexate dose for all patients. BCC basal cell carcinoma, CI confidence interval, CMM cutaneous malignant melanoma, cSCC cutaneous squamous cell carcinoma, MTX methotrexate, OR odds ratio.

Among the BCC cases, 1214 (0.9%) were exposed to MTX with a cumulative dose ≥2.5 g compared with 8389 (0.6%) of controls, yielding a minimally adjusted OR (95% CI) of 1.45 (1.37–1.54). In the fully adjusted analysis, the OR was 1.29 (1.20–1.38). The OR increased with increasing cumulative dose (Table 2 and Fig. 1). The association was not modified markedly by age, sex or BCC localisation (Table 3).

**Table 2:** Risk of basal cell carcinoma, cutaneous squamous cell carcinoma and cutaneous malignant melanoma according to the cumulative dose of methotrexate.

Subgroup	Cases, *n*	Controls, *n*	Crude OR^a^ (95% CI)	Adjusted OR^b^ (95% CI)
*BCC*				
Non-use	128,631	1,292,298	1.0 (ref.)	1.0 (ref.)
Ever-use	2816	22,146	1.28 (1.23–1.33)	1.16 (1.11–1.22)
Accumulated dose ≥2.5 g	1214	8389	1.45 (1.37–1.54)	1.29 (1.20–1.38)
Cumulative dose (g)				
0–1.25	1080	9707	1.12 (1.05–1.19)	1.03 (0.96–1.10)
1.25–2.5	522	4050	1.30 (1.18–1.42)	1.20 (1.09–1.31)
2.5–5.0	583	4348	1.35 (1.24–1.47)	1.24 (1.13–1.36)
5.0–7.5	322	2138	1.52 (1.35–1.70)	1.39 (1.24–1.58)
≥7.5	309	1903	1.63 (1.45–1.84)	1.48 (1.31–1.68)
*cSCC*				
Non-use	18,164	183,374	1.0 (ref.)	1.0 (ref.)
Ever-use	497	3224	1.56 (1.42–1.72)	1.22 (1.09–1.37)
Accumulated dose ≥2.5 g	233	1296	1.82 (1.58–2.10)	1.61 (1.37–1.89)
Cumulative dose (g)				
0–1.25	182	1366	1.35 (1.15–1.58)	0.99 (0.84–1.17)
1.25–2.5	82	562	1.47 (1.17–1.86)	1.15 (0.90–1.46)
2.5–5.0	106	623	1.72 (1.40–2.11)	1.47 (1.18–1.83)
5.0–7.5	62	311	2.02 (1.54–2.66)	1.81 (1.36–2.41)
≥7.5	65	362	1.82 (1.40–2.37)	1.57 (1.18–2.08)
*CMM*				
Non-use	25,617	256,736	1.0 (ref.)	1.0 (ref.)
Ever-use	451	3944	1.15 (1.04–1.27)	1.19 (1.06–1.33)
Accumulated dose ≥2.5 g	183	1465	1.25 (1.07–1.46)	1.35 (1.13–1.61)
Cumulative dose (g)				
0–1.25	193	1776	1.09 (0.94–1.27)	1.13 (0.97–1.32)
1.25–2.5	75	703	1.07 (0.84–1.36)	1.10 (0.86–1.41)
2.5–5.0	97	747	1.30 (1.05–1.61)	1.37 (1.10–1.72)
5.0–7.5	47	378	1.25 (0.92–1.69)	1.31 (0.96–1.79)
≥7.5	39	340	1.15 (0.83–1.60)	1.21 (0.85–1.71)

*BCC* basal cell carcinoma, *CI* confidence interval, *CMM* cutaneous malignant melanoma, *cSCC* cutaneous squamous cell carcinoma, *OR* odds ratio.

^a^Adjusted for age, sex and calendar time (by design).

^b^Adjusted for age, sex, calendar time and other covariates (see 'Covariates').

**Table 3:** Effect modification of the risk of basal cell carcinoma, cutaneous squamous cell carcinoma, and cutaneous malignant melanoma associated with a cumulated dose of methotrexate ≥2.5 g compared to never-use.

Subgroup	Cases exposed/unexposed	Controls exposed/unexposed	Crude OR^a^ (95% CI)	Adjusted OR^b^ (95% CI)	*P* value for interaction
*BCC*					
All	1214/128,631	8389/1,292,298	1.5 (1.4–1.5)	1.3 (1.2–1.4)	
Sex					0.43
Male	429/60,463	2885/606,820	1.5 (1.3–1.7)	1.3 (1.2–1.5)	
Female	785/68,168	5504/685,478	1.4 (1.3–1.5)	1.3 (1.2–1.4)	
Age					0.32
<65 years	368/55,058	2415/552,530	1.5 (1.4–1.7)	1.3 (1.2–1.5)	
65–75 years	462/40,465	3386/406,630	1.4 (1.2–1.5)	1.2 (1.1–1.3)	
≥75 years	384/33,108	2588/333,138	1.5 (1.3–1.7)	1.3 (1.2–1.5)	
Localisation					<0.0001
Head and neck	458/48,659	3485/488,581	1.3 (1.2–1.5)	1.2 (1.1–1.3)	
Trunk	176/24,406	1487/244,100	1.2 (1.0–1.4)	1.0 (0.9–1.2)	
Upper limb	67/6124	381/61,549	1.8 (1.4–2.3)	1.5 (1.2–2.0)	
Lower limb	73/5121	379/51,583	1.9 (1.5–2.5)	1.7 (1.3–2.2)	
Other/unspecified	440/44,321	2657/446,485	1.7 (1.5–1.8)	1.5 (1.3–1.6)	
*cSCC*					
All	233/18,164	1296/183,374	1.8 (1.6–2.1)	1.6 (1.4–1.9)	
Sex					0.001
Male	121/10,131	523/102,292	2.3 (1.9–2.8)	2.1 (1.7–2.6)	
Female	112/8033	773/81,082	1.5 (1.2–1.8)	1.3 (1.0–1.6)	
Age					<0.0001
<65 years	18/3124	156/31,427	1.2 (0.7–1.9)	1.0 (0.6–1.6)	
65–75 years	65/5429	462/54,880	1.4 (1.1–1.8)	1.2 (0.9–1.6)	
≥75 years	150/9611	678/97,067	2.3 (1.9–2.7)	2.0 (1.7–2.5)	
Localisation					<0.0001
Head and neck	107/8305	578/83,734	1.9 (1.5–2.3)	1.7 (1.4–2.1)	
Trunk	15/1617	143/16,268	1.0 (0.6–1.8)	1.0 (0.6–1.7)	
Upper limb	16/2097	167/21,019	1.0 (0.6–1.6)	0.8 (0.4–1.3)	
Lower limb	16/1335	109/13,400	1.5 (0.9–2.5)	1.3 (0.7–2.2)	
Other/unspecified	79/4810	299/48,953	2.8 (2.1–3.5)	2.3 (1.8–3.1)	
*CMM*					
All	183/25,617	1465/256,736	1.3 (1.1–1.5)	1.3 (1.1–1.6)	
Sex					0.63
Male	71/11,759	533/117,830	1.3 (1.0–1.7)	1.4 (1.1–.8)	
Female	112/13,858	932/138,906	1.2 (1.0–1.5)	1.3 (1.1–1.6)	
Age					0.14
<65 years	64/15,315	546/153,265	1.2 (0.9–1.5)	1.2 (0.9–1.6)	
65–75 years	57/5985	520/59,991	1.1 (0.8–1.4)	1.2 (0.9–1.6)	
≥75 years	62/4317	399/43,480	1.6 (1.2–2.1)	1.7 (1.3–2.2)	
Localisation					0.003
Head and neck	38/2785	175/28,142	2.3 (1.6–3.2)	2.4 (1.7–3.5)	
Trunk	78/10,896	572/109,297	1.4 (1.1–1.7)	1.5 (1.1–1.9)	
Upper limb	28/3757	273/37,583	1.0 (0.7–1.5)	1.1 (0.7–1.6)	
Lower limb	29/6017	352/59,994	0.8 (0.6–1.2)	0.9 (0.6–1.3)	
Other/unspecified	10/2162	93/21,720	1.1 (0.6–2.1)	1.2 (0.6–2.3)	

*BCC* basal cell carcinoma, *CI* confidence interval, *CMM* cutaneous malignant melanoma, *cSCC* cutaneous squamous cell carcinoma, *OR* odds ratio.

^a^Adjusted for age, calendar time (by design).

^b^Adjusted for age, sex, calendar time and other covariates (see 'Covariates').

### cSCC

A total of 233 (1.2%) cSCC cases and 1296 (0.7%) controls had been exposed to MTX with a cumulative dose ≥2.5 g. This resulted in a minimally adjusted OR of 1.82 (1.58–2.10). After adjustment, the OR was 1.61 (1.37-1.89). A noticeable dose–response pattern was observed (Table 2 and Fig. 1). The association was more pronounced among individuals ≥75 years and in males (Table 3).

### CMM

Among the CMM cases, 183 (0.7%) were exposed to MTX with a cumulative dose ≥2.5 g compared with 1465 (0.6%) of controls, yielding a minimally adjusted OR of 1.25 (1.07–1.46). In the fully adjusted analysis, the OR was 1.35 (1.13–1.61). However, no evidence of dose response was observed (Table 2 and Fig. 1). The overall association was more pronounced for CMMs located in the head and neck region (Table 3).

**Fig. 2:**
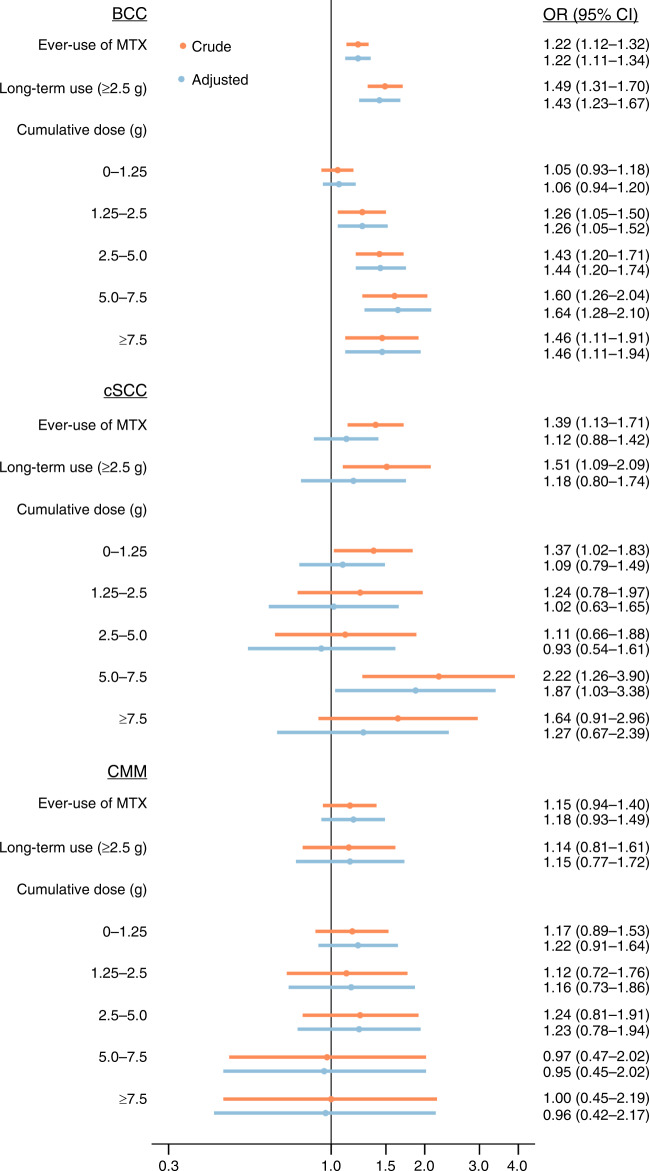
Risk of basal cell carcinoma, cutaneous squamous cell carcinoma, and cutaneous malignant melanoma according to cumulative methotrexate dose restricted to patients with psoriasis. BCC basal cell carcinoma, CI confidence interval, CMM cutaneous malignant melanoma, cSCC cutaneous squamous cell carcinoma, MTX methotrexate, OR odds ratio.

The findings of the primary analysis were not substantially affected by varying the lag period, however, the association for cSCC moved towards the null with increasing lag period, e.g., from 1.61 (1.37–1.89) with 12 months of lag to 1.44 (CI 1.17–1.78) with 60 months of lag (Supplementary Table 2). Results were largely unaffected by omitting patients with any exposure to MTX in the time period 1995–1996 (Supplementary Table 3). In the analysis restricted to patients with psoriasis, the OR for the use of MTX (cumulative dose ≥2.5 g) associated with BCC, SCC and CMM was 1.43 (1.23–1.67), 1.18 (0.80–1.74) and 1.15 (0.77–1.72), respectively (Fig. 2 and Supplementary Tables 4 and 5). The covariates that, individually, had the largest effect on the association between MTX and cSCC were azathioprine and sulfasalazine, where the minimally adjusted ORs of 1.82 (1.58–2.10) was attenuated to 1.68 (1.45–1.93) and 1.68 (1.44–1.97), respectively, upon adjustment (Supplementary Table 6).

## Discussion

In this study, use of a cumulative MTX dose ≥2.5 g was associated with an increased risk of BCC, cSCC and CMM compared with no use of MTX. For BCC and cSCC a dose–response association was observed. However, the association with CMM and cSCC disappeared when restricting the study population to patients with psoriasis, indicating that surveillance bias influenced our results.

Previous studies have shown conflicting findings. Most notably, CIRT included 2391 patients (median age 66 years; 19% females) randomised to low-dose MTX (mean dosage 14.9 mg weekly) and 2395 patients (median age 66 years; 18% females) randomised to placebo. Risk of skin cancer was increased in the MTX-treated group (2.2%) compared with the placebo (1.1%) with a HR (95% CI) of 2.1 (1.3–3.3). When stratifying by skin cancer type, the HR was 1.4 (0.7–2.7) for BCC, 3.3 (1.6–6.7) for cSCC, and 2.0 (0.5–8.0) for CMM [3, 4]. In contrast to this study, confounding or surveillance bias is less likely given the randomised nature of CIRT; however, skin cancer was not a prespecified endpoint of interest, and the CIs were wide.

Observational studies have reported increased risks as well as neutral associations for MTX associated with NMSC. In an Australian cohort of 405 patients with psoriatic arthritis and rheumatoid arthritis, MTX ever-use was associated with a higher risk of NMSC compared to no MTX usage [standardised incidence ratio (SIR) 4.6, 95% CI 0.7–33.2]. The risk increase was present in BCC (SIR 3.0, 95% CI 2.4–3.8) and cSCC (SIR 1.6, 95% CI 1.6–3.4), but only with an apparent dose–response association for BCC [22]. An American cohort study, including 6841 patients with rheumatoid arthritis, reported an enhanced risk for a second NMSC for individuals with MTX use ≥1 year compared with no use (adjusted HR 1.2, 95% CI 1.0–1.5) [23]. Moreover, an investigation including a cohort of 7955 patients with psoriasis from several countries demonstrated a risk increase associated with ever-use of MTX for BCC (HR 8.6, 95% CI 3.3–22.4) but not cSCC (HR 1.3, 95% CI 0.4–4.2). However, no data on a dose–response association was reported [24]. In a recent Swedish case–control study nested within a cohort of psoriasis patients, ever-use of MTX was associated with cSCC (OR 1.2, 95% CI 1.0–1.5) in crude analyses; however, after adjusting for use of immunosuppressants, other than MTX, the association was close to unity (OR 1.1, 95% CI 0.9–1.3) [25].

With regards to CMM, previous investigations have demonstrated positive as well as neutral associations. An Australian cohort study of patients with rheumatoid arthritis reported a SIR of CMM of 3.0 (95% CI 1.2–6.2) associated with the ever-use of MTX [26]. In a recent Norwegian nationwide case–control study, use of ≥4 dispensed prescriptions of MTX was associated with an increased risk of CMM compared with those with ≤1 prescription (rate ratio 1.27, 95% CI 1.04–1.55) [27]. However, in a nationwide Swedish cohort study, ever-use of MTX was only weakly associated with CMM (HR 1.2, 95% CI 1.1–1.3) and subsequent analyses found no evidence of a dose–response pattern [28, 29]. In a Swedish case–control study nested in a cohort of psoriasis patients, no association between MTX ever-use and CMM was observed (OR 1.0, 95% CI 0.8–1.3) [30].

In a recent systematic review and meta-analysis, including 12 investigations and 16,642 cases of melanoma, individuals with MTX use had a small increased risk of melanoma compared to non-users (pooled relative risk, 1.15; 95% CI, 1.08–1.22) [31].

The specific indications for MTX treatment warrants discussion as the indications themselves might be associated with increased skin cancer risk. In a recent meta-analysis including patients with psoriasis, the pooled relative risk was 2.2 (95% CI 1.3–3.5) for cSCC and 1.3 (95% CI 0.7–2.3) for BCC. For severe psoriasis, the corresponding figures were 11.7 (95% CI 1.5–90.7) and 3.2 (95% CI 1.3–7.6) [32]. In a Danish cohort study, an increased risk for NMSC was observed for patients with mild (adjusted IRR 1.7, 95% CI 1.6–1.8) and severe psoriasis (IRR 1.3, 95% CI 1.1–1.6) compared with the Danish general population [33]. Since MTX is used to treat severe psoriasis, a potential risk factor for NMSC and CMM [32, 34, 35], we cannot exclude residual confounding by indication. Further, users of MTX may be more likely to undergo skin examinations and diagnostic workup compared with never-users, potentially leading to surveillance bias. In our supplementary analysis nested in psoriasis patients, the association with MTX remained only for BCC, which provides evidence of surveillance bias and/or confounding by indication in the main analyses. However, the CIs were wide in this supplementary analysis. Data on sun exposure were not available in the registries used and we cannot exclude confounding by UV exposure—the most important environmental risk factor for NMSC. Finally, our investigation was conducted among people born in any of the Nordic countries, where skin types I to III are most prevalent. This must be considered when extrapolating the findings to populations with more diverse skin types.

To summarise, we observed evidence of a dose-dependent increase in risks of BCC and cSCC, but not CMM, associated with use of MTX. However, the observed associations were of limited magnitude and supplementary analyses suggested that confounding and surveillance bias played a role. At present, our findings therefore cannot support that skin cancer risk should be an important consideration when prescribing MTX. However, our findings do deserve further attention for future investigations that would ideally include data on UV exposure.

## Supplementary information

STROBE- checklist

Supplemental material

## Data Availability

The datasets generated and/or analysed during this study are available from the corresponding author upon reasonable request.
