# Supplementary material for: Use of methotrexate and risk of skin cancer: a nationwide case–control study
Source: Br J Cancer. 2023 Feb 4;128(7):1311–9. doi: 10.1038/s41416-023-02172-7 (PMC10050200; doi:10.1038/s41416-023-02172-7)
Supplement: Supplementary file 1 — STROBE- checklist [file 41416_2023_2172_MOESM1_ESM.pdf]

STROBE Statement—Checklist of items that should be included in reports of ***case-control studies***

	Item No	Recommendation	
**Title and abstract**	1	(*a*) Indicate the study's design with a commonly used term in the title or the abstract	Title
		(*b*) Provide in the abstract an informative and balanced summary of what was done and what was found	page 2
**Introduction**			
Background/rationale	2	Explain the scientific background and rationale for the investigation being reported	page 2
Objectives	3	State specific objectives, including any prespecified hypotheses	pages 3-4
**Methods**			
Study design	4	Present key elements of study design early in the paper	pages 3-4
Setting	5	Describe the setting, locations, and relevant dates, including periods of recruitment, exposure, follow-up, and data collection	page 3
Participants	6	(*a*) Give the eligibility criteria, and the sources and methods of case ascertainment and control selection. Give the rationale for the choice of cases and controls	page 4
		(*b*) For matched studies, give matching criteria and the number of controls per case	page 4
Variables	7	Clearly define all outcomes, exposures, predictors, potential confounders, and effect modifiers. Give diagnostic criteria, if applicable	page 4-5
Data sources/ measurement	8*	For each variable of interest, give sources of data and details of methods of assessment (measurement). Describe comparability of assessment methods if there is more than one group	page 4
Bias	9	Describe any efforts to address potential sources of bias	page 4-5
Study size	10	Explain how the study size was arrived at	Page 6 and Supplementary figure
Quantitative variables	11	Explain how quantitative variables were handled in the analyses. If applicable, describe which groupings were chosen and why	page 4
Statistical methods	12	(*a*) Describe all statistical methods, including those used to control for confounding	page 4-5
		(*b*) Describe any methods used to examine subgroups and interactions	page 4-5
		(*c*) Explain how missing data were addressed	N/A
		(*d*) If applicable, explain how matching of cases and controls was addressed	Page 4
		(*e*) Describe any sensitivity analyses	page 5-6 & Supplementary material
**Results**			
Participants	13*	(a) Report numbers of individuals at each stage of study—eg numbers potentially eligible, examined for eligibility, confirmed eligible, included in the study, completing follow-up, and analysed	Page 6 and Supplementary figure
		(b) Give reasons for non-participation at each stage	Page 6 and Supplementary figure
		(c) Consider use of a flow diagram	Page 6 and Supplementary figure
Descriptive data	14*	(a) Give characteristics of study participants (eg demographic, clinical, social) and information on exposures and potential confounders	Page 6 & Table 1
		(b) Indicate number of participants with missing data for each variable of interest	Table 1
Outcome data	15*	Report numbers in each exposure category, or summary measures of exposure	Table 2
Main results	16	(*a*) Give unadjusted estimates and, if applicable, confounder-adjusted estimates and their precision (eg, 95% confidence interval). Make clear which confounders were adjusted for and why they were included	Table 2
		(*b*) Report category boundaries when continuous variables were categorized	Table 2
		(*c*) If relevant, consider translating estimates of relative risk into absolute risk for a meaningful time period	-

Other analyses	17	Report other analyses done—eg analyses of subgroups and interactions, and sensitivity analyses	page 7 and Supplementary Tables 2-5
**Discussion**			
Key results	18	Summarise key results with reference to study objectives	page 7
Limitations	19	Discuss limitations of the study, taking into account sources of potential bias or imprecision. Discuss both direction and magnitude of any potential bias	page 8
Interpretation	20	Give a cautious overall interpretation of results considering objectives, limitations, multiplicity of analyses, results from similar studies, and other relevant evidence	page 8-9
Generalisability	21	Discuss the generalisability (external validity) of the study results	page 8
**Other information**			
Funding	22	Give the source of funding and the role of the funders for the present study and, if applicable, for the original study on which the present article is based	N/A

*Give information separately for cases and controls.

Note: An Explanation and Elaboration article discusses each checklist item and gives methodological background and published examples of transparent reporting. The STROBE checklist is best used in conjunction with this article (freely available on the Web sites of PLoS Medicine at http://www.plosmedicine.org/, Annals of Internal Medicine at http://www.annals.org/, and Epidemiology at http://www.epidem.com/). Information on the STROBE Initiative is available at http://www.strobe-statement.org.
