# Supplementary material for: Use of methotrexate and risk of skin cancer: a nationwide case–control study
Source: Br J Cancer. 2023 Feb 4;128(7):1311–9. doi: 10.1038/s41416-023-02172-7 (PMC10050200; doi:10.1038/s41416-023-02172-7)
Supplement: Supplementary file 2 — Supplemental material [file 41416_2023_2172_MOESM2_ESM.pdf]

Supplementary material

Title: Use of methotrexate and risk of skin cancer: a nationwide case-control study

Sam Polesie[1,2], Martin Gillstedt[1,2], Sigrún Alba Jóhannesdóttir Schmidt[3,4], Alexander Egeberg[5], Anton Pottegård[6], and Kasper Kristensen[6]

[1] Department of Dermatology and Venereology, Institute of Clinical Sciences, Sahlgrenska Academy, University of Gothenburg, Gothenburg, Sweden.

[2] Region Västra Götaland, Sahlgrenska University Hospital, Department of Dermatology and Venereology, Gothenburg, Sweden.

[3] Department of Clinical Epidemiology, Aarhus University Hospital, Aarhus, Denmark

[4] Department of Dermatology, Aarhus University Hospital, Aarhus, Denmark.

[5] Department of Dermatology, Bispebjerg Hospital, University of Copenhagen, Denmark.

[6] Clinical Pharmacology, Pharmacy and Environmental Medicine, Department of Public Health, University of Southern Denmark, Odense, Denmark.

Correspondence to:

Sam Polesie, MD, PhD

Gröna stråket 16,

SE-413 45 Gothenburg, Sweden

Email: sam.polesie@gu.se

Attachments: 7

Supplementary Appendix: 1

Supplementary Figure: 1

Supplementary Tables: 5

Supplementary Appendix – Danish Nationwide Health Registries

All Danish residents have universal access to healthcare and are assigned a unique civil registration number which enable reliable linking of healthcare databases and registers.

For this investigation, data from the five registers/databases were used: *the Danish National Prescription Registry*; *the Danish National Patient Registry*, *the Danish Cancer Registry; the Population Education Registry* and *The Danish Civil Registration System*.

The Danish National Prescription Registry includes data on all dispensed prescription drugs by Danish residents since 1995. The data include the type of drug, date of dispensation, and quantity. The dosing text and the specific indication for prescription are not available. Moreover, drugs prescribed in the inhospital setting are unavailable. All included drugs are classified according to the Anatomic Therapeutic Chemical (ATC) index. The quantity dispensed for each prescription is described by the number and strength of the pharmaceutical entities (e.g., tablets), and defined daily doses (DDD), respectively.

The Danish National Patient Registry includes nationwide data on all non-psychiatric hospital admissions since 1977 and on ambulatory hospital contacts and psychiatric admissions since 1995. Discharge/contact diagnoses have been coded according to International Classification of Diseases, (ICD) 8th revision (ICD-8) from 1977 to 1993 and ICD-10 since 1994.

The Danish Cancer Registry includes incident cases of cancer on a nationwide basis since 1943 and provides accurate and almost complete records of cancer cases in Denmark. Cancer diagnoses are classified according to the ICD-10 and the ICD for Oncology (ICD-O-3) for topography and morphology.

The Population Education Registry contains information on nearly all adult Danish citizens. The registry provides the highest completed level of education, which is defined as the longest duration of schooling.

The Danish Civil Registration System contains data on addresses, migration, and date of death.

Supplementary Fig. – Final case population

BCC, basal cell carcinoma; CMM, cutaneous malignant melanoma; cSCC, cutaneous squamous cell carcinoma

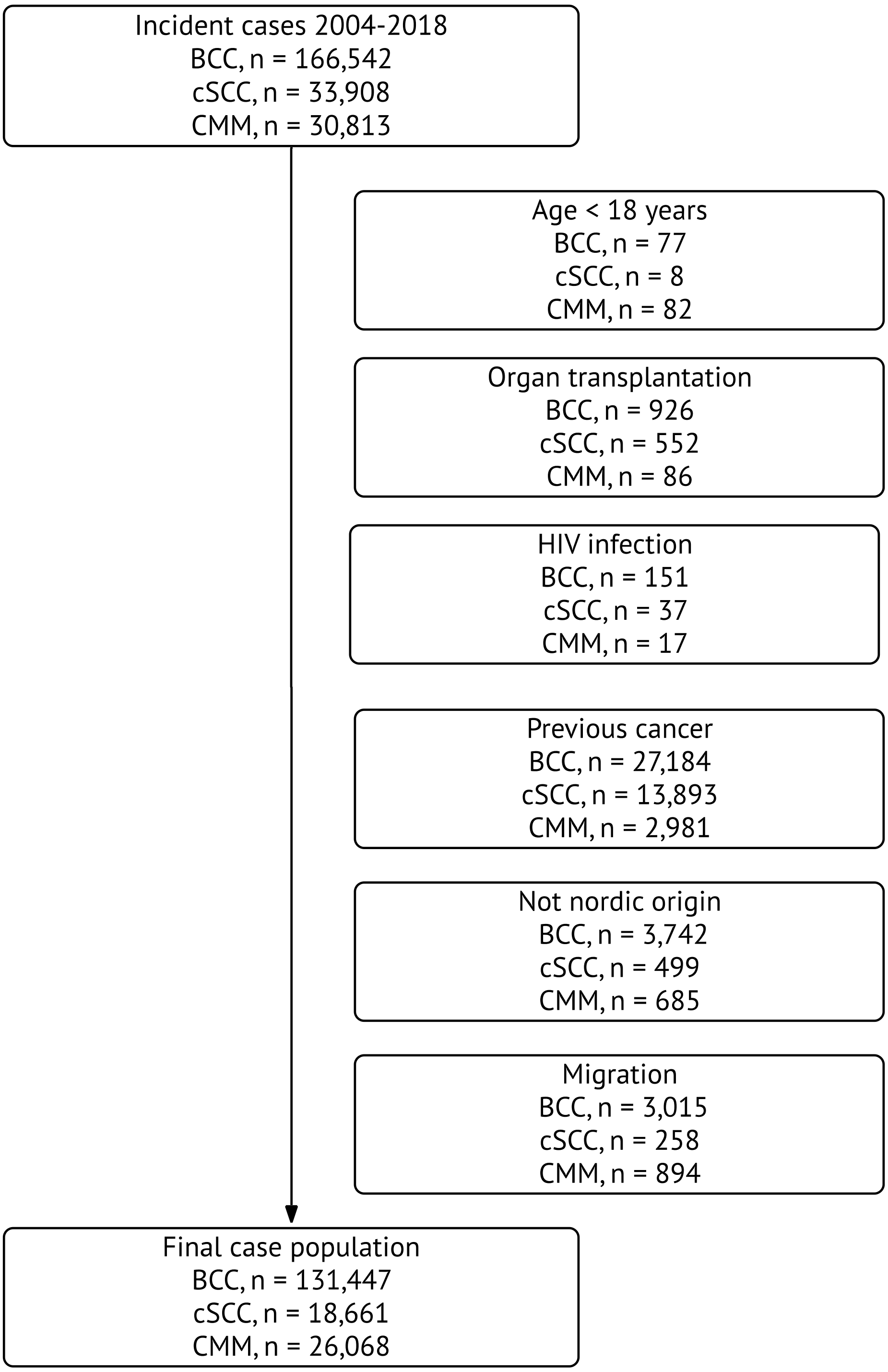

Supplementary Tables

Supplementary Table 1 – Definition of outcome and exposure variables**.**

**Cancer definitions**		
BCC	*ICD-10*	C00, C440-C449
	Morphology code	8090/3 BCC, NOS<br>8091/3 BCC, multifocal, superficial<br>8092/3 BCC, infiltrating<br>8093/3 BCC, fibroepithelial<br>8097/3 BCC, nodular<br>8123/3 BCC, basaloid carcinoma
cSCC	*ICD-10*	C00, C440-C449
	Morphology code	8051/3 SCC, verrucous carcinoma<br>8052/3 SCC, papillary<br>8070/3 SCC, NOS<br>8070/6 SCC, metastatic<br>8071/3 SCC, keratinizing, NOS<br>8072/3 SCC, large cell, non-keratinizing, NOS<br>8073/3 SCC, squamous cell carcinoma, small cell<br>8074/3 SCC, spindle cell<br>8075/3 SCC, adenoid<br>8076/3 SCC, microinvasive<br>8078/3 SCC, with horn formation<br>8083/3 SCC, basaloid<br>8084/3 SCC, clear cell type<br>8094/3 mixed basal-SCC<br>8095/3 metatypical carcinoma
CMM	*ICD-10*	C430-C439
	Morphology code	8720/3 Malignant melanoma, NOS<br>8721/3 Nodular melanoma<br>8730/3 Amelanotic melanoma<br>8740/3 Melanoma in junctional nevus<br>8741/3 Melanoma in precan. melanosis<br>8742/3 Lentigo melanoma<br>8743/3 Superficial spreading melanoma<br>8744/3 Acral melanoma<br>8745/3 Desmoplastic melanoma<br>8760/3 Melanoma in composite nevus<br>8761/3 Melanoma in giant pigmented nevus<br>8772/3 Spindle cell melanoma<br>8780/3 Malignant blue nevus<br>8790/3 Malignant cellular blue nevus
**Exclusion criteria**		
Any cancer	*ICD10 diagnosis group*	11 - 301 for cSCC and BCC<br>12 – 168 excl. 62 for CMM

	(c_diaggr_icd_10) from the Danish Cancer registry	
Organ transplant	*ICD-10*	Z94 (except Z945 and Z947)
	NCSP-code	KFQA, KFQB, KGDG, KJJC, KJLE, KKAS
HIV infection	*ICD-10*	B20-24 and Z21
**Use of drugs**		
Methotrexate	*ATC code*	L04AX03
	SKS codes	BWHA115 or ML04AX03 and at least one of the following supplementary codes: BWDB0, BWAA
Leflunomide	*ATC code*	L04AA13
Azathioprine	*ATC code*	L04AX01
Sulfasalazine	*ATC code*	A07EC01
Cyclosporine	*ATC code*	L04AD01
Tumor necrosis factor α inhibitors	*ATC code*	L04AB
	SKS codes	ML04AB, BOHJ18A
Interleukin pathway-inhibitors	*ATC code*	L04AC,
	SKS codes	ML04AC, BOHJ18B
Hydrochlorothiazide	*ATC code*	C03AA03, C03AB03, C03AX01, C09DX03, C03EA, C09BA, C09DA, C07B, C09DX01, C09XA52, C09XA54 (excluding C07BA06, C09BA04)
Topical and oral retinoids	*ATC code*	D10AD, D05BB, D10BA01
Photosensitizing antibiotics (tetracycline, macrolides, fluoroquinolones, and aminoquinolines)	*ATC code*	J01AA07, J01FA, J01MA, P01BA
PUVA treatment	*NSCP code*	BNGA1
	ATC code	D05BA
**Presumed indication for methotrexate**		
Rheumatoid arthritis	*ICD-10*	M05-M06
Psoriasis and/or psoriatic arthritis	*ICD-10*	L40, M070-M073
	ATC	D05AX
Inflammatory bowel disease	*ICD-10*	K50 K51 K528C M074-M076
Atopic dermatitis	*ICD-10*	L20
Dermatitis (unspecified)	*ICD-10*	L309

ATC, Anatomical Therapeutic Chemical; BCC, basal cell carcinoma; CMM, cutaneous malignant melanoma cSCC; cutaneous squamous cell carcinoma; DDD, Defined daily doses; ICD-10, International Classification of Diseases, Tenth Revision; NCSP, Nordic Classification of Surgical Procedures; NOS; Not otherwise specified; SKS, catalog of administrative and clinical codes used in the Danish health care system (In Danish Sygehusvæsenets klassifikationssystem).

**Medical history**		
Alcohol-associated conditions	*ICD-10*	E244, E529A, G312, G621, G721, I426, F10, K292, K70, K860, T519, Z502, Z714, Z721
	ATC code	N07BB
Diabetes	*ICD-10*	E10-E14
	ATC code	A10
Chronic obstructive pulmonary disease	*ICD-10*	J42-J44
	ATC code	R03BB
Peptic ulcer (including gastric and duodenal ulcer)	*ICD-10*	K25-K28
Ischemic heart disease or congestive heart failure	*ICD-10*	I20–I25, I110, I130, I132, I42, I43, I50, I517
Kidney disease	*ICD-10*	E102, E112, E122, E132, E142, I12–13 (excluding I12.9), N01, N03, N083, N085, N118C, N14, N150, N16, N18 (excluding N181), N19, N26, P960, Q601, Q602, Z992
**Anatomical site of cSCC or BCC**		
Skin of head and neck	*ICD-10*	C00, C440-C444
Skin of trunk	*ICD-10*	C445
Skin of upper limb, incl. shoulder	*ICD-10*	C446
Skin of lower limb, incl. hip	*ICD-10*	C447
Unspecified part of skin	*ICD-10*	C448-C449
**Anatomical site of CMM**		
Skin of head and neck	*ICD-10*	C430-C434
Skin of trunk	*ICD-10*	C435
Skin of upper limb, incl. shoulder	*ICD-10*	C436
Skin of lower limb, incl. hip	*ICD-10*	C437
Unspecified part of skin	*ICD-10*	C438-C439
**Education**		
None or basic education (~ 9-10 years)	*National classification of educational programs, Statistics Denmark (Uddannelsesniveau)*	10-20
High school or vocational training (~ 10 – 12 years)		30-50
Higher education (~ 13 years and above)		60-80
Unknown		-

Supplementary Table 2 – Effect of varying lag time on the association between use of methotrexate (cumulative dose ≥2.5 g) and basal cell carcinoma, cutaneous squamous cell carcinoma, and cutaneous malignant melanoma

**BCC**		**cSCC**		**CMM**	
**Lag time (months)**	**Adjusted OR (95% CI)**	**Lag time (months)**	**Adjusted OR (95% CI)**	**Lag time (months)**	**Adjusted OR (95% CI)**
0	1.30 (1.21-1.39)	0	1.63 (1.40-1.90)	0	1.28 (1.08-1.52)
6	1.31 (1.22-1.40)	6	1.61 (1.38-1.89)	6	1.33 (1.12-1.58)
12*	1.29 (1.20-1.38)	12*	1.61 (1.37-1.89)	12*	1.35 (1.13-1.61)
18	1.28 (1.19-1.37)	18	1.58 (1.34-1.87)	18	1.32 (1.10-1.58)
24	1.29 (1.20-1.39)	24	1.59 (1.34-1.89)	24	1.31 (1.08-1.58)
30	1.29 (1.20-1.40)	30	1.60 (1.34-1.91)	30	1.31 (1.08-1.59)
36	1.26 (1.16-1.36)	36	1.60 (1.33-1.92)	36	1.33 (1.09-1.62)
42	1.25 (1.15-1.36)	42	1.64 (1.36-1.98)	42	1.30 (1.06-1.60)
48	1.22 (1.12-1.33)	48	1.57 (1.30-1.91)	48	1.31 (1.06-1.62)
54	1.23 (1.12-1.34)	54	1.44 (1.17-1.76)	54	1.32 (1.06-1.64)
60	1.24 (1.13-1.35)	60	1.44 (1.17-1.78)	60	1.31 (1.04-1.64)

* A lag time of 12 months corresponds to our main analysis.

BCC, basal cell carcinoma; CI, confidence interval; CMM; cutaneous malignant melanoma; cSCC, cutaneous squamous cell carcinoma; OR, odds ratio.

Supplementary Table 3 – New-user design excluding all persons from the study base who redeemed a prescription for MTX in the time period 1995–1996.

	Cases, n	Controls, n	Crude OR (95% CI)	Adjusted OR (95% CI)
BCC				
Non-use	128,631	1,292,298	1.0 (ref.)	1.0 (ref.)
Ever use	2,492	19,743	1.27 (1.22-1.32)	1.16 (1.10-1.21)
Long-term use (≥ 2.5 g)	1,001	6,956	1.44 (1.35-1.54)	1.28 (1.18-1.38)
Cumulative dose (g)				
]0-1.25[	1,004	9,031	1.12 (1.05-1.19)	1.03 (0.96-1.10)
[1.25 - 2.5[	487	3,756	1.30 (1.19-1.43)	1.20 (1.09-1.33)
[2.5 - 5.0[	532	3,866	1.38 (1.26-1.52)	1.27 (1.16-1.40)
[5.0 - 7.5[	251	1,755	1.44 (1.26-1.65)	1.32 (1.15-1.52)
≥ 7.5	218	1,335	1.64 (1.42-1.90)	1.49 (1.28-1.73)
cSCC				
Non-use	18,164	183,374	1.0 (ref.)	1.0 (ref.)
Ever use	410	2,854	1.45 (1.31-1.61)	1.15 (1.01-1.29)
Long-term use (≥ 2.5 g)	181	1,063	1.73 (1.47-2.02)	1.54 (1.28-1.85)
Cumulative dose (g)				
]0-1.25[	159	1,267	1.27 (1.08-1.50)	0.94 (0.79-1.13)
[1.25 - 2.5[	70	524	1.34 (1.05-1.73)	1.06 (0.81-1.37)
[2.5 - 5.0[	92	562	1.65 (1.33-2.06)	1.44 (1.14-1.82)
[5.0 - 7.5[	47	247	1.92 (1.41-2.63)	1.72 (1.24-2.38)
≥ 7.5	42	254	1.68 (1.21-2.33)	1.47 (1.04-2.08)
CMM				
Non-use	25,617	256,736	1.0 (ref.)	1.0 (ref.)
Ever use	404	3,537	1.15 (1.03-1.27)	1.19 (1.06-1.35)
Long-term use (≥ 2.5 g)	155	1,204	1.29 (1.09-1.53)	1.40 (1.15-1.69)
Cumulative dose (g)				
]0-1.25[	179	1,672	1.07 (0.92-1.25)	1.12 (0.96-1.32)
[1.25 - 2.5[	70	661	1.06 (0.83-1.36)	1.11 (0.86-1.43)
[2.5 - 5.0[	89	646	1.38 (1.11-1.72)	1.46 (1.15-1.84)
[5.0 - 7.5[	37	310	1.20 (0.85-1.69)	1.25 (0.88-1.78)
≥ 7.5	29	248	1.17 (0.80-1.72)	1.24 (0.83-1.85)

BCC, basal cell carcinoma; CI, confidence interval; CMM; cutaneous malignant melanoma; cSCC, cutaneous squamous cell carcinoma; OR, Odds ratio.

Supplementary Table 4 – Patient characteristics for the supplementary analysis restricted to patients with psoriasis.

	**BCC Cases**	**BCC Controls**	**cSCC Cases**	**cSCC Controls**	**CMM Cases**	**CMM Controls**
All	(n=5,062)	(n=50,576)	(n=772)	(n=7,696)	(n=831)	(n=8,310)
Age, median (IQR)	67 (59-75)	67 (59-75)	75 (66-81)	75 (66-81)	64 (51-73)	64 (51-73)
Male sex	2,238 (44.2%)	22,369 (44.2%)	401 (51.9%)	3,994 (51.9%)	370 (44.5%)	3,700 (44.5%)
Use of methotrexate						
Ever use	752 (14.9%)	6,344 (12.5%)	121 (15.7%)	901 (11.7%)	124 (14.9%)	1,103 (13.3%)
≥ 2.5 g	282 (5.6%)	1,963 (3.9%)	44 (5.7%)	302 (3.9%)	39 (4.7%)	353 (4.2%)
Drug use						
Retinoids	295 (5.8%)	2,909 (5.8%)	74 (9.6%)	433 (5.6%)	42 (5.1%)	500 (6.0%)
Photosensitizing antibiotics	3,482 (68.8%)	33,533 (66.3%)	533 (69.0%)	5,030 (65.4%)	559 (67.3%)	5,557 (66.9%)
Hydroclorothiazide	897 (17.7%)	9,123 (18.0%)	228 (29.5%)	1,615 (21.0%)	115 (13.8%)	1,308 (15.7%)
PUVA treatment	94 (1.9%)	898 (1.8%)	21 (2.7%)	158 (2.1%)	14 (1.7%)	148 (1.8%)
Leflunomide	13 (0.3%)	103 (0.2%)	(n<5)	7 (0.1%)	5 (0.6%)	16 (0.2%)
Azathioprine	86 (1.7%)	799 (1.6%)	31 (4.0%)	116 (1.5%)	15 (1.8%)	149 (1.8%)
Cyclosporine	67 (1.3%)	555 (1.1%)	18 (2.3%)	74 (1.0%)	(n<5)	81 (1.0%)
Sulfasalazine	283 (5.6%)	2,437 (4.8%)	54 (7.0%)	329 (4.3%)	46 (5.5%)	409 (4.9%)
TNFi or ILi	122 (2.4%)	943 (1.9%)	20 (2.6%)	127 (1.7%)	28 (3.4%)	222 (2.7%)
Medical history						
Rheumatoid arthirtis	147 (2.9%)	1,405 (2.8%)	34 (4.4%)	216 (2.8%)	32 (3.9%)	238 (2.9%)
Inflammatory bowel disease	116 (2.3%)	861 (1.7%)	17 (2.2%)	124 (1.6%)	13 (1.6%)	146 (1.8%)
Atopic dermatitis	15 (0.3%)	191 (0.4%)	(n<5)	19 (0.2%)	(n<5)	32 (0.4%)
Unspecified dermatitis	68 (1.3%)	751 (1.5%)	16 (2.1%)	141 (1.8%)	10 (1.2%)	153 (1.8%)
Alcohol associated conditions	243 (4.8%)	3,131 (6.2%)	41 (5.3%)	453 (5.9%)	30 (3.6%)	540 (6.5%)
Diabetes	514 (10.2%)	6,245 (12.3%)	127 (16.5%)	1,159 (15.1%)	78 (9.4%)	906 (10.9%)
COPD	374 (7.4%)	4,529 (9.0%)	95 (12.3%)	868 (11.3%)	43 (5.2%)	632 (7.6%)
Kidney disease	85 (1.7%)	954 (1.9%)	44 (5.7%)	198 (2.6%)	16 (1.9%)	136 (1.6%)
Peptic ulcer	143 (2.8%)	1,606 (3.2%)	30 (3.9%)	280 (3.6%)	16 (1.9%)	237 (2.9%)
Ischemic heart disease or congestive heart failure	728 (14.4%)	7,301 (14.4%)	152 (19.7%)	1,580 (20.5%)	99 (11.9%)	1,005 (12.1%)
Education						
Short	1,395 (27.6%)	17,182 (34.0%)	268 (34.7%)	2,716 (35.3%)	205 (24.7%)	2,489 (30.0%)
Medium	2,289 (45.2%)	21,876 (43.3%)	338 (43.8%)	3,132 (40.7%)	414 (49.8%)	3,821 (46.0%)
Long	1,255 (24.8%)	10,246 (20.3%)	118 (15.3%)	1,426 (18.5%)	195 (23.5%)	1,799 (21.6%)
Unknown	123 (2.4%)	1,272 (2.5%)	48 (6.2%)	422 (5.5%)	17 (2.0%)	201 (2.4%)

BCC, basal cell carcinoma; CMM; cutaneous malignant melanoma; COPD, chronic obstructive pulmonary disease; cSCC, cutaneous squamous cell carcinoma; ILi, interleukin pathway-inhibitors; IQR, interquartile range; OR, Odds ratio; PUVA, psoralen plus ultraviolet light A; TNFi, tumor necrosis factor α inhibitors.

Supplementary Table 5 – Risk of basal cell carcinoma, cutaneous squamous cell carcinoma, and malignant melanoma according to cumulative dose of methotrexate when restricting to patients with psoriasis.

	**Cases**	**Control**	**Crude OR (95% CI)**	**Adjusted OR (95% CI)**
BCC				
Non-use	4,310	44,232	1.0 (ref.)	1.0 (ref.)
Ever use	752	6,344	1.22 (1.12-1.32)	1.22 (1.11-1.34)
Long-term use (≥ 2.5 g)	282	1,963	1.49 (1.31-1.70)	1.43 (1.23-1.67)
Cumulative dose (g)				
]0-1.25[	329	3,226	1.05 (0.93-1.18)	1.06 (0.94-1.20)
[1.25 - 2.5[	141	1,155	1.26 (1.05-1.50)	1.26 (1.05-1.52)
[2.5 - 5.0[	144	1,036	1.43 (1.20-1.71)	1.44 (1.20-1.74)
[5.0 - 7.5[	77	495	1.60 (1.26-2.04)	1.64 (1.28-2.10)
≥ 7.5	61	432	1.46 (1.11-1.91)	1.46 (1.11-1.94)
cSCC				
Non-use	651	6,795	1.0 (ref.)	1.0 (ref.)
Ever use	121	901	1.39 (1.13-1.71)	1.12 (0.88-1.42)
Long-term use (≥ 2.5 g)	44	302	1.51 (1.09-2.09)	1.18 (0.80-1.74)
Cumulative dose (g)				
]0-1.25[	56	426	1.37 (1.02-1.83)	1.09 (0.79-1.49)
[1.25 - 2.5[	21	173	1.24 (0.78-1.97)	1.02 (0.63-1.65)
[2.5 - 5.0[	16	148	1.11 (0.66-1.88)	0.93 (0.54-1.61)
[5.0 - 7.5[	15	71	2.22 (1.26-3.90)	1.87 (1.03-3.38)
≥ 7.5	13	83	1.64 (0.91-2.96)	1.27 (0.67-2.39)
CMM				
Non-use	707	7,207	1.0 (ref.)	1.0 (ref.)
Ever use	124	1,103	1.15 (0.94-1.40)	1.18 (0.93-1.49)
Long-term use (≥ 2.5 g)	39	353	1.14 (0.81-1.61)	1.15 (0.77-1.72)
Cumulative dose (g)				
]0-1.25[	63	550	1.17 (0.89-1.53)	1.22 (0.91-1.64)
[1.25 - 2.5[	22	200	1.12 (0.72-1.76)	1.16 (0.73-1.86)
[2.5 - 5.0[	24	197	1.24 (0.81-1.91)	1.23 (0.78-1.94)
[5.0 - 7.5[	8	84	0.97 (0.47-2.02)	0.95 (0.45-2.02)
≥ 7.5	7	72	1.00 (0.45-2.19)	0.96 (0.42-2.17)

BCC, basal cell carcinoma; CI, confidence interval; CMM; cutaneous malignant melanoma; cSCC, cutaneous squamous cell carcinoma; OR, Odds ratio.

Supplementary Table 6 – Analysis of all covariates including the odds ratio for use of methotrexate (cumulative dose ≥2.5 g) when adjusting for each individual covariate, the odds ratio for the covariate associated with basal cell carcinoma, cutaneous squamous cell carcinoma, or cutaneous malignant melanoma, and the prevalence of the covariate in exposed (cumulative methotrexate dose ≥2.5 g) controls and unexposed (never users of methotrexate) controls.

	Adjusted OR[1]	OR for covariate associated with outcome	Covariate prevalence in exposed controls, n (%)	Covariate prevalence in unexposed controls, n (%)
**BCC**				
Minimally adjusted[2]	1.45 (1.37-1.54)	-	-	-
Fully adjusted[3]	1.29 (1.20-1.38)	-	-	-
Photosensitizing drugs				
Retinoids	1.44 (1.36-1.53)	1.46 (1.40-1.52)	243 (2.9)	16,136 (1.2)
Photosensitizing antibiotics	1.40 (1.32-1.49)	1.23 (1.22-1.25)	6,326 (75.4)	695,046 (53.8)
Hydroclorothiazide	1.45 (1.37-1.54)	1.04 (1.02-1.06)	1,530 (18.2)	182,577 (14.1)
PUVA treatment	1.45 (1.37-1.54)	1.13 (0.97-1.32)	83 (1.0)	1,119 (0.1)
Immunosuppresive drugs				
Leflunomide	1.45 (1.36-1.54)	1.31 (1.07-1.59)	394 (4.7)	62 (0.0)
Azathioprine	1.41 (1.33-1.50)	1.50 (1.41-1.59)	569 (6.8)	6,240 (0.5)
Cyclosporine	1.44 (1.36-1.53)	1.40 (1.21-1.63)	275 (3.3)	676 (0.1)
Sulfasalazine	1.37 (1.28-1.46)	1.22 (1.16-1.27)	4,034 (48.1)	9,667 (0.7)
TNFi or ILi	1.36 (1.28-1.46)	1.51 (1.38-1.66)	1,410 (16.8)	761 (0.1)
Medical history				
Alcohol	1.45 (1.36-1.54)	0.71 (0.69-0.73)	257 (3.1)	57,509 (4.5)
Diabetes	1.46 (1.37-1.55)	0.80 (0.78-0.82)	960 (11.4)	108,589 (8.4)
COPD	1.46 (1.37-1.55)	0.86 (0.83-0.88)	827 (9.9)	76,150 (5.9)
Kidney disease	1.45 (1.37-1.54)	0.97 (0.92-1.02)	142 (1.7)	15,917 (1.2)
Peptic ulcer	1.46 (1.37-1.55)	0.87 (0.84-0.91)	399 (4.8)	33,700 (2.6)
Ischemic heart disease or congestive heart failure	1.45 (1.37-1.55)	0.98 (0.96-0.99)	1,268 (15.1)	149,185 (11.5)
Education				
Highest achieved education	1.47 (1.39-1.57)	-	-	-
**cSCC**				
Minimally adjusted	1.82 (1.58-2.10)	-	-	-
Fully adjusted	1.61 (1.37-1.89)	-	-	-
Photosensitizing drugs				
Retinoids	1.77 (1.54-2.04)	2.12 (1.87-2.40)	44 (3.4)	1,326 (0.7)
Photosensitizing antibiotics	1.75 (1.52-2.02)	1.25 (1.21-1.29)	979 (75.5)	96,540 (52.6)
Hydroclorothiazide	1.85 (1.61-2.13)	1.70 (1.64-1.76)	236 (18.2)	33,119 (18.1)
PUVA treatment	1.81 (1.57-2.08)	2.19 (1.58-3.04)	11 (0.8)	156 (0.1)
Immunosuppresive drugs				
Leflunomide	1.82 (1.57-2.10)	1.88 (1.15-3.08)	56 (4.3)	9 (0.0)
Azathioprine	1.68 (1.45-1.93)	3.56 (3.17-4.00)	61 (4.7)	859 (0.5)
Cyclosporine	1.75 (1.52-2.02)	3.17 (2.32-4.33)	30 (2.3)	86 (0.0)
Sulfasalazine	1.68 (1.44-1.97)	1.42 (1.27-1.59)	595 (45.9)	1,436 (0.8)
TNFi or ILi	1.73 (1.49-2.01)	2.04 (1.59-2.60)	186 (14.4)	86 (0.0)
Medical history				
Alcohol	1.82 (1.58-2.10)	0.98 (0.90-1.06)	37 (2.9)	6,711 (3.7)
Diabetes	1.82 (1.58-2.09)	1.17 (1.11-1.22)	155 (12.0)	19,619 (10.7)
COPD	1.82 (1.58-2.09)	1.18 (1.12-1.24)	135 (10.4)	14,760 (8.0)
Kidney disease	1.82 (1.58-2.09)	1.64 (1.50-1.79)	33 (2.5)	3,574 (1.9)
Peptic ulcer	1.81 (1.58-2.09)	1.11 (1.03-1.20)	96 (7.4)	6,883 (3.8)
Ischemic heart disease or congestive heart failure	1.82 (1.58-2.09)	1.10 (1.06-1.15)	256 (19.8)	32,013 (17.5)
Education				
Highest achieved education	1.82 (1.58-2.10)	-	-	-
**CMM**				
Minimally adjusted	1.25 (1.07-1.46)	-	-	-
Fully adjusted	1.35 (1.13-1.61)	-	-	-
Photosensitizing drugs				
Retinoids	1.25 (1.07-1.46)	1.21 (1.12-1.31)	49 (3.3)	5,729 (2.2)
Photosensitizing antibiotics	1.23 (1.06-1.44)	1.11 (1.08-1.14)	1,108 (75.6)	143,482 (55.9)
Hydroclorothiazide	1.25 (1.07-1.46)	1.15 (1.11-1.20)	250 (17.1)	28,146 (11.0)
PUVA treatment	1.25 (1.07-1.46)	1.17 (0.80-1.69)	13 (0.9)	205 (0.1)
Immunosuppresive drugs				
Leflunomide	1.28 (1.09-1.50)	0.96 (0.57-1.63)	79 (5.4)	10 (0.0)
Azathioprine	1.25 (1.07-1.46)	1.08 (0.93-1.27)	92 (6.3)	1,277 (0.5)
Cyclosporine	1.27 (1.08-1.48)	0.72 (0.46-1.13)	57 (3.9)	134 (0.1)
Sulfasalazine	1.31 (1.11-1.55)	1.00 (0.89-1.12)	718 (49.0)	1,756 (0.7)
TNFi or ILi	1.27 (1.08-1.50)	1.14 (0.92-1.41)	311 (21.2)	246 (0.1)
Medical history				
Alcohol	1.25 (1.07-1.46)	0.64 (0.59-0.69)	46 (3.1)	11,967 (4.7)
Diabetes	1.26 (1.08-1.47)	0.92 (0.87-0.97)	174 (11.9)	17,868 (7.0)
COPD	1.27 (1.08-1.48)	0.71 (0.66-0.76)	134 (9.1)	11,499 (4.5)
Kidney disease	1.25 (1.07-1.46)	1.03 (0.91-1.17)	23 (1.6)	2,558 (1.0)
Peptic ulcer	1.26 (1.08-1.47)	0.82 (0.75-0.91)	70 (4.8)	5,129 (2.0)
Ischemic heart disease or congestive heart failure	1.26 (1.08-1.47)	0.92 (0.88-0.97)	220 (15.0)	22,646 (8.8)
Education				
Highest achieved education	1.28 (1.09-1.49)	-	-	-

[1] The OR for methotrexate use (cumulative dose ≥2.5 g) associated with BCC, cSCC, or CMM adjusted for age, sex, and calendar time (by design) and the covariate of interest

[2]Adjusted for age, sex, and calendar time (by design).

[3]Adjusted for age, sex, calendar time and all covariates mentioned in this tableBCC, basal cell carcinoma; CI, confidence interval; CMM; cutaneous malignant melanoma; cSCC, cutaneous squamous cell carcinoma; OR, Odds ratio.
